# Mediterranean Diet Adherence and Dietary Attitudes in Patients with Inflammatory Bowel Disease

**DOI:** 10.3390/nu12113429

**Published:** 2020-11-08

**Authors:** Josip Vrdoljak, Marino Vilović, Piero Marin Živković, Ivana Tadin Hadjina, Doris Rušić, Josipa Bukić, Josip Anđelo Borovac, Joško Božić

**Affiliations:** 1 Department of Pathophysiology, University of Split School of Medicine, 21000 Split, Croatia; j.vrdoljak9@gmail.com (J.V.); marino.vilovic@mefst.hr (M.V.); piero.zivkovic@gmail.com (P.M.Ž.); josip.borovac@me.com (J.A.B.); 2 Department of Endocrinology, Diabetes and Metabolic Diseases, University Hospital of Split, 21000 Split, Croatia; 3 Department of Gastroenterology, University Hospital of Split, 21000 Split, Croatia; ihtadina@gmail.com; 4 Department of Pharmacy, University of Split School of Medicine, 21000 Split, Croatia; doris.rusic@mefst.hr (D.R.); josipa.bukic@mefst.hr (J.B.); 5 Institute of Emergency Medicine of Split-Dalmatia County, 21000 Split, Croatia

**Keywords:** Mediterranean diet, inflammatory bowel disease, Mediterranean Diet Service Score (MDSS), nutrition

## Abstract

A specific diet regimen is a promising way of managing inflammatory bowel disease (IBD), with the Mediterranean diet (MD) being a likely candidate due to its potential to modulate gut inflammation. Therefore, the aim of this study was to investigate nutritional habits and dietary attitudes of IBD patients, and to assess their adherence to the Mediterranean diet. The study enrolled 50 Crohn’s disease and 44 ulcerative colitis patients, with clinical and laboratory parameters taken. Dietary attitudes were examined, and adherence to MD was assessed using the Mediterranean Diet Service Score (MDSS). Average MDSS score was 6.0 (5.0–7.0), while only nine participants fulfilled criteria for Mediterranean diet adherence. Moreover, all of them were men (*p* = 0.021). Low percentage of adherence to recommended guidelines was observed for eating olive oil (25.5%), fresh fruit (14.9%), and vegetables (10.6%). Significant positive correlation was observed between total MDSS points and high-density lipoprotein (HDL) cholesterol levels (*p* = 0.002). The majority of the patients (86.2%) considered that a more controlled diet could reduce their IBD symptoms, while 17% visited a nutritionist for diet advice. The majority of patients (84%) would visit educational programs regarding nutrition. In conclusion, adherence to MD was very low, while IBD patients were willing to extend their nutritional knowledge if proper educational programs were organized.

## 1. Introduction

Inflammatory bowel disease (IBD) is a chronic autoimmune disorder that is characterized by inflammation of the gastrointestinal tract. Two major types of IBD are ulcerative colitis (UC) and Crohn’s disease (CD) [1]. The prevalence of IBD continues to grow because of young onset age and low mortality [2]. Moreover, due to its growing prevalence and numerous systemic complications, it is likely that IBD will become a major health problem in the future [3,4,5].

The precise pathomechanism of IBD remains unclear. A combination of susceptible genes, inappropriate diet, and an inappropriate immune response leads to a breakdown of intestinal homeostasis [6,7]. Since IBD is a chronic disease, treating it and retaining a durable remission poses a big challenge in today’s gastroenterology. There is a constantly expanding multitude of therapeutics to treat IBD, such as immunosuppressants and biological drugs like infliximab and adalimumab [8]. Some patients become refractory to these pharmacological agents, in which case they are in need of surgery [9]. Furthermore, diet plays a big role in the clinical care of patients with IBD [10]. Many studies have shown that clinical remission and mucosal healing can be achieved with a formula-defined enteral feed [11]. However, the mechanisms for this response are still unclear. Current theories hypothesize that these dietary remissions are attained because of the changes in gut microbiota, improved nutritional status, avoidance of additives, or reduced allergenic potential to gut content [11,12,13].

IBD patients often wonder whether proper and precise diet can change the course of their disease, while, with the right diet modification patterns, they have a potential pathway to manage their illness [14]. A study performed by Casanova et al. found that 77% of patients avoid certain foods during remission, while 86% of patients with active disease go onto stricter diets [15]. Moreover, patients often go onto self-prescribed diets, many of which lack strong scientific evidence, including the Paleolithic diet, gluten-free diet, low-FODMAP (fermentable oligosaccharides, disaccharides, monosaccharides, and polyols) diet, or specific carbohydrate-based diet [16]. Strict diet and avoidance of certain foods can even lead to malnutrition, as well as vitamin and/or other deficiencies [15]. Current evidence points to the Western diet as one of the main culprits attributing to the rising IBD incidence [7,13,17]. A high ratio of ω-6 to ω-3, high sugar and animal protein, and high dietary additive content are all characteristic components of a Western-style of diet, all of which lead to gut dysbiosis and an increased IBD risk [13].

The Mediterranean diet (MD) is a characteristic dietary pattern established amid the olive tree-growing areas of the Mediterranean [18]. The traditional Mediterranean diet is defined by high consumption of vegetables, fruits, olive oil, nuts, and legumes, as well as fish and unprocessed cereals, low intake of meat and meat products, and low intake of dairy products (other than long-lasting cheeses). Alcohol consumption was also fairly common in the traditional Mediterranean, mostly as a glass of red wine during meals [18]. The MD could have an impact on various aspects of life in the general population, in different age groups. Studies report that the MD is associated with better health-related quality of life in elderly people [19], but low MD adherence has shown significant connection with high perceived loneliness and high stress levels in adolescence [20].

Furthermore, current literature has some new and promising data concerning the effects of the Mediterranean diet on IBD. A study by Khalili et al. found that greater adherence to the Mediterranean diet was associated with a significantly lower risk of later-onset CD [21]. Moreover, a recent study by Papada et al. showed an association between the MedDiet score and improved quality of life in a population of CD patients [22]. Furthermore, Lo et al. provided evidence of a significant association between healthy lifestyle, including MD adherence, and reduced mortality in IBD patients [23], while Eder et al. consider the MD to be most beneficial for elderly IBD patients [24]. Overall, the MD is considered to have a high potential to modulate gut inflammation and to be a therapeutic and preventive tool for IBD [25,26].

Considering the proposed important dietary impact on IBD patients and preventive potential regarding the Mediterranean diet, the main goal of this cross-sectional study was to gather data about the nutritional habits and dietary attitudes of prediagnosed IBD patients and to assess their adherence to the Mediterranean diet using the Mediterranean Diet Service Score (MDSS).

## 2. Materials and Methods 

### 2.1. Study Design and Population

This cross-sectional study enrolled 44 patients with ulcerative colitis and 50 patients with Crohn’s disease diagnosed at the Department of Gastroenterology, University Hospital of Split between December 2017 and April 2019. The diagnosis of IBD was set according to recent guidelines by the European Crohn’s and Colitis Organization and the European Society of Gastrointestinal and Abdominal Radiology [27]. All participants were informed about the procedures and goals of this study. Prior to the beginning, the study protocol was approved by the Ethics Committees of the University of Split School of Medicine and the University Hospital of Split. Patients gave written informed consent, and all procedures were carried out in correspondence with the 1964 Declaration of Helsinki and its later modifications.

All subjects were aged between 18 and 65 years, with IBD diagnosed at least for 1 year. Furthermore, patients which had one of the following exclusion criteria were not eligible to continue the study protocol and, therefore, were excluded from the study: patients with diabetes, malignancies, or severe chronic renal, cardiac, or endocrine diseases that resulted in specific dietary change as part of the therapy.

### 2.2. Clinical and Laboratory Parameters

Detailed medical and specific gastrointestinal anamnesis was taken from each of the subjects, including data taken from medical history records. In such a way, information regarding therapeutics, extra-intestinal complications, and nutritional deficiencies was assessed. Furthermore, an experienced gastroenterologist performed clinical examination, with assessment of anthropometrical measurements according to standardized protocols.

After a 12 h fast, all subjects underwent venous blood sampling. Lipid panel parameters, as well as total protein and albumin levels, were assessed using standard biochemical procedures, while high-sensitivity C-reactive protein (hsCRP) levels were measured via latex turbidimetric (Abbott Laboratories, Chicago, IL, USA).

Finally, within 3 days of blood sampling, stool samples were collected as well, for determination of fecal calprotectin (FC) levels. A turbidimetric immunoassay was used via a Buhlmann fCAL turbo assay (Buhlmann Laboratories AG, Schonenbuch, Switzerland).

### 2.3. Disease Activity Parameters

For disease activity assessment, several different methods were used by the same eligible gastroenterologist, specific for Crohn’s disease or UC. In Crohn’s disease patients, endoscopic evaluation for disease activity was assessed with a simple endoscopic score for Crohn’s disease (SES-CD). Ulcers, total affected and ulcerated surface, and narrowing characteristics were evaluated. The following empirical cutoff values in the final results were used: inactive disease ≤ 2; mild activity 2–7; moderate activity 7–16; severe disease ≥ 16 [28]. Quantitative scores for Crohn’s disease activity included Crohn’s disease activity index (CDAI), which is a well-established mathematical index that determines the current severity of Crohn’s disease based on signs and symptoms in the past 7 days. CDAI threshold scores for analysis were as follows: clinical remission < 150; mild to moderate activity 150–450; severe disease > 450 [29].

For UC endoscopic evaluation, the ulcerative colitis endoscopic index of severity (UCEIS) was used. UCEIS is a quantitative indicator of mucosal inflammation established on colonoscopic findings. Disease stage is organized as remission (0–1), mild (2–4), moderate (5–6), or severe (7–8) [30]. Finally, Mayo score/disease activity index (Mayo/DAI) was used for UC patients as well, which is a clinical score that assesses UC activity. The Mayo/DAI score provides a good correspondence with UC severity: <2, remission; 3–5, mild disease; 6–10, moderate disease; 10–12, severe disease [31]. As latest guidelines suggest caution in using clinical indices due to possible lack of proper validation, SES-CD and UCEIS scores were used for stratification of patients into groups with different disease activity [32].

### 2.4. Dietary Habits and Attitudes

All included patients were given a questionnaire that examined specific information and attitudes regarding their disease and eating habits. The questionnaire was made at the Department of Pathophysiology by two experienced gastroenterologists after a detailed literature search.

The first part of the questionnaire consisted of 12 questions and was focused on the disease, as well as its symptoms and attitudes regarding diet. The questions included were related to time from IBD diagnosis, therapy, surgery as a part of treatment, stoma presence, consumption of nutritional support supplements, intravenous nutritional support in the last 12 months, and self-assessment of the disease severity. Furthermore, the questionnaire examined digestive disturbances that IBD patients connect with specific food groups, and attitudes regarding IBD and nutrition in general. Therefore, collected information included food groups that patients connected with IBD-related symptoms, digestive problems that occur in those situations, micronutrient deficiencies, and patients’ information source and their rating of nutritional information.

The second part of the questionnaire was the MDSS [33], a validated questionnaire established on the current update of the Mediterranean Diet Pyramid [34], which assesses adherence to individual food groups of MD and total MD adherence. Specific eating patterns of 14 different food groups are assessed with MDSS, and scores are given only if eating guidelines are followed. A score of 3 is awarded to those individuals who meet the desired consumption frequency of foods such as fruits, vegetables, cereals (bread, breakfast cereals, rice, pasta), and olive oil. A score of 2 is given to those who meet the recommended consumption of nuts and dairy products. Lastly, a score of 1 is given to those who meet the recommendations for potatoes, legumes, eggs, fish, white meat, red meat, sweets, and fermented beverages (wine and beer).

In such a way, food that should be a part of every meal in MD is given the most points, followed by food groups preferred for daily and weekly consummation [33]. A score of 0 is given when the number of servings does not meet the recommendations. Therefore, the score can range from 0 to 24 points, and the cutoff value for the definition of Mediterranean Diet adherence is set at >13.5 points.

### 2.5. Statistical Analysis

Statistical analysis was performed with statistical software MedCalc, version 19.1.2. (MedCalc Software, Ostend, Belgium). Categorical variables were presented as whole numbers and percentages, while chi-square test and Fisher’s exact test were used for testing differences between groups. Normality of data distribution for continuous variables was assessed with a D’Agostino–Pearson test, and data were presented in the form of mean and standard deviation or median and interquartile range. Therefore, differences in anthropometric and laboratory parameters between groups were assessed with a *t*-test for independent samples, while differences in disease duration, hsCRP, FC, and total MDSS score were tested with Mann–Whitney test. Correlation analysis of total MDSS score and other parameters was performed with Spearman’s coefficient of rank correlation. Statistical significance was considered at *p* < 0.05.

## 3. Results

### 3.1. Baseline Clinical and Laboratory Characteristics

This study enrolled a total of 94 IBD patients, of which 44 were in the UC group and 50 were in the CD group. The groups did not significantly differ according to gender (*p* = 0.756), disease duration (*p* = 0.426), or age (*p* = 0.101). Waist circumference (*p* = 0.016) and BMI (*p* = 0.017) were significantly larger in the UC group in comparison to CD patients, while IBD-associated operations and extraintestinal manifestations occurred significantly more often in the CD group compared to the UC group (*p* < 0.001 and *p* = 0.002, respectively). Lastly, nutritional support was also used more often in the CD group (*p* = 0.048). Detailed information regarding clinical and anthropometric parameters of the study population can be seen in Table 1.

Laboratory analysis showed that the UC group had significantly higher levels of total cholesterol (5.4 ± 1.43 vs. 4.3 ± 1.26 mmol/L; *p* < 0.001), low-density lipoprotein (LDL) cholesterol (3.38 ± 1.27 vs. 2.35 ± 0.89 mmol/L; *p* < 0.001), high-density lipoprotein (HDL) cholesterol (1.5 ± 0.48 vs. 2.35 ± 0.89 mmol/L; *p* = 0.016), and albumins (40.9 ± 4.6 vs. 38.3 ± 5.2 g/L; *p* = 0.008) when compared with the CD group. Furthermore, laboratory disease activity parameters such as hsCRP and fecal calprotectin showed no significant difference between the groups (*p* = 0.601 and *p* = 0.128, respectively). According to SES-CD and UCEIS scores, most of the patients had moderate disease activity (39.4%), followed by severe disease activity (31.9%), mild activity (17%), and remission (11.7%), without significant differences between the groups. Detailed laboratory and disease activity parameters (Table 2).

### 3.2. Mediterranean Diet and Individual Food Group Adherence

Considering individual food group preferences from the scope of Mediterranean diet, the participants in both UC and CD groups most commonly adhered to recommended guidelines for eating potatoes (64.9%) and sweets (64.9%), followed by cereals (52.1%) and red meat (51.1%). A lower percentage of adherence was observed for olive oil (25.5%), fresh fruit (14.9%), and vegetables (10.6%). The CD group had a significantly higher percentage of adherence for eating milk and dairy products (36% vs. 13.6%, *p* = 0.025) and sweets (76% vs. 52.3%, *p* = 0.028) in comparison with UC patients. The average MDSS score of entire population was 6.0 (5.0–7.0), while only nine participants fulfilled criteria for Mediterranean diet adherence (Table 3). Moreover, all patients that adhered to the diet were men (*p* = 0.021).

Furthermore, when patient population was divided into two groups according to disease activity (remission/mild endoscopic disease (*n* = 27) and moderate/severe endoscopic disease (*n* = 67)), there were no significant differences between adherence to any of the individual food groups (*p* > 0.05). Moreover, there were no differences between the mentioned groups in total MDSS score (6.0 (5.0–7.75) vs. 6.0 (5.0–7.0); *p* = 0.598) and total adherence to Mediterranean diet (11.1% vs. 9.0%; *p* = 0.713).

### 3.3. Food and IBD Symptoms

A total of 81 (86.2%) participants considered that certain food groups exacerbate their digestive symptoms associated with IBD. Most commonly, patients associate that with spicy food (68.1%), milk (54.3%), sodas (54.3%), and large meals (51.1%). UC patients considered sweets to be significantly more associated with IBD symptoms in comparison to CD patients (50% vs. 28%; *p* = 0.048), while there were no significant differences between the UC and CD patients for all the other investigated food groups (Table 4).

The digestive problems that most often occurred after ingesting suspected food were stomach pain (64.9%) and diarrhea (51.1%), while symptoms with lesser occurrence were in the form of heartburn, vomiting, blood in stool, and frequent bowel movements. Blood in stool after ingesting suspected food types was significantly more reported in the UC group when compared to CD patients (29.5% vs. 8%; *p* = 0.008), as well as frequent bowel movements (27.3% vs. 10.0%; *p* = 0.035) (Table 5).

Correlation analysis was performed between total MDSS points and different biochemical and anthropometric parameters. Results showed a significant positive correlation between total MDSS points and HDL cholesterol levels (*r* = 0.312; *p* = 0.002). Other parameters did not significantly correlate with total MDSS score (Table 6). 

### 3.4. Attitudes Regarding IBD and Nutrition

Lastly, we investigated patients’ dietary attitudes about IBD and nutrition. Most of them (90.4%) considered that proper nutrition has an important role in their illness and in life in general (97.9%). Moreover, a majority of the patients (86.2%) considered that a better and more controlled diet could reduce their IBD symptoms. A total of 90.4% of the patients got their diet information from their physician, while 17% visited a nutritionist for diet advice, and only 14.9% used the internet to inform themselves about the diet related to their illness. UC patients considered educational programs on nutrition to be beneficial in this population significantly more than CD patients (100% vs. 84%; *p* = 0.006) (Table 7).

## 4. Discussion

This study presents adherence to MD guidelines using MDSS in IBD patients, as well as individual food group intake differences in CD and UC patients in a traditional Mediterranean setting. Dietary patterns and attitudes regarding the disease and nutrition were also assessed.

Considering the frequency of certain food group consumption in the framework of MDSS, this IBD population mostly followed adherence guidelines for potatoes (64.9%), sweets (64.9%), cereals (52.1%), and red meat (51.1%). Interestingly, food groups that are a hallmark of the MD and should be consumed in every meal, such as fresh fruit and vegetables, had a very poor adherence rate (14.9% and 10.6%, respectively). Furthermore, total adherence to MD was rather low (9.6%), and it could mean that further Westernization of the diet took place in our population, where the MD is traditional and was widely consumed in recent generations [7]. These results correspond with the findings of Taylor et al., whose IBD participants also had low Predimed Mediterranean Diet adherence scores [35], pointing to the continuation of the diet industrialization trend as showed by Garcia Closas et al. [36]. Interestingly, all of our participants that fulfilled the criteria for MD adherence were men.

Olive oil plays a central part in the Mediterranean diet pyramid, and its importance was shown in multiple studies [37,38]. Olive oil reduces chronic inflammation in the dextran sulfate sodium (DSS)-colitis rat model, it leads to peroxisome proliferator activated-receptor (PPAR-γ) upregulation and nuclear factor kappa B (NF-κB) and mitogen activated protein kinase (MAPK) signaling pathway inhibition [37]. Furthermore, a study on cardiovascular patients determined a significant reduction in plasma concentration of hsCRP, interleukin 6 (IL-6), and tumor necrosis factor (TNF) in patients who followed a MD supplemented with extra virgin olive oil [38]. Furthermore, Cariello et al. showed that extra-virgin olive oil reduced histopathological evidence of intestinal inflammation in a mouse model of induced colitis [39]. In our study, olive oil was consumed in the recommended frequency by 25.5% of patients, which is considerably more compared to the participants from the study conducted by Taylor et al. (6%) [33]. However, considering that our study is based in the Mediterranean area, where olive oil is expected to have a traditionally large impact on daily dietary patterns, this difference is not surprising, and it could be seen as a surprisingly low result.

Another part of the MD that has traditional value is wine consumption. Studies show that phenolic compounds present in wine have some efficacy in preventing IBD and colitis-associated colorectal cancer [40,41]. Moreover, in a study by Swanson et al., where they evaluated the effect of short-term moderate wine drinking on inactive IBD, it was shown that moderate wine drinking delays long-term risk for disease relapse [42]. Nevertheless, caution is advised since greater alcohol portions lead to an increase in oxidative stress and inflammation of the gut [43]. As wine is traditionally consumed in this area, fermented beverage adherence was surprisingly low (only 6.4% met the recommendations). In consideration of wine benefits, proposed in previous studies, moderate wine consumption could be advised to IBD patients with inactive disease.

In a recent study by Papada et al., where MD adherence in CD patients via MedDiet score was investigated, the authors exhibited a significantly higher score in patients with inactive CD in comparison with patients with active disease [22]. Although they did show that CD patients in remission are more likely to follow the MD, they had no cutoff values to define the patients as adherent or nonadherent to the MD. On the other hand, in our study, MDSS with its clear cutoff values showed no difference in adherence between the patients who were in remission/mild disease group and those who were in the moderate/severe disease group.

In our study, we further show a significant positive correlation between total MDSS points and HDL cholesterol levels (*p* = 0.002). Similar findings were reported by Penalvo et al. in a recent cohort study, as those participants who adhered to MD had significantly higher HDL cholesterol levels and a healthier ratio of triglyceride to HDL cholesterol [44]. These findings further confirm the positive effects of MD, especially olive oil, whose influences are ameliorative to lipid profile and, consequently, cardiovascular diseases in general [45].

The majority of patients (68.1%) associated spicy food ingestion with exacerbation of IBD symptoms, followed by milk (54.4%) and sodas (54.4%). Our finding correlates with a study by Aggarwal et al., in which the spicy food group was also most commonly connected with IBD symptom worsening (41% of participants) [46], while Tomar et al. showed that 84.8% of their IBD population chose to exclude spicy food to prevent disease relapse [47]. This connection could be present due to the inflammatory effects of specific vegetable compounds present in spicy food, but the direct pathophysiological link remains unclear and should be further investigated [48]. These results lead us to another potential positive aspect of MD, as representative food groups do not have an emphasis on spicy ingredients. Furthermore, it is known that milk is associated with IBD symptom worsening as a consequence of its lactose content, while sodas also provide extra fermented carbohydrate content, as well as artificial sweeteners, which were also shown to change the gut microbiota [49].

Interestingly, our questionnaire results showed that sweets were connected with IBD symptom aggravation significantly more in UC patients when compared with the CD group (50.0% vs. 28.0%; *p* = 0.048). Moreover, CD patients followed MD eating guidelines for sweets in a significantly higher percentage than UC group (52.3% vs. 76.0%; *p* = 0.028). These results imply that sweets are a possible important food group that should be avoided in IBD patients, as it could be a trigger for symptom worsening. Furthermore, the MD puts sweets as a food group that should be consumed in a rare manner, and patients who follow adherence guidelines could avoid exacerbations due to sweet consumption. When fermentable carbohydrates are consumed in large quantities, the intestines’ absorptive ability can be exceeded, in turn leading to increased gut permeability and inflammation [7]. Nevertheless, evidence for a connection between sugar consumption and IBD onset is mixed, as some studies showed no clear connection between them [50], while others showed a significant association between high sugar consumption and IBD development [51,52]. Furthermore, Racine et al. showed a positive association between high sugar and soft drink consumption with increased UC risk (incidence rate ratios for the fifth versus first quintile, 1.68 (1.00–2.82); *p* = 0.02) [53]. Since fermentation occurs in the colon, it would be logical to assume that fermentable carbohydrate overconsumption would largely lead to increased inflammation of the colon and, hence, have a possible bigger impact on ulcerative colitis onset.

We also reported patients’ dietary attitudes about IBD and nutrition, as well as their sources of information about dieting. While most of our participants got their dietary information from their physician, only 17% of them visited a licensed nutritionist. In a study by Tomar et al., around 45% of participants received dietary counseling [47]. However, they did not specify whether counseling was made by a physician, nutritionist, or someone else. Furthermore, 90.4% of patients believe that proper nutrition has a large role in IBD, and 86.2% believe that a more controlled diet could reduce IBD symptoms. The majority of participants (91.5%) believe that educational programs about nutrition could be beneficial to IBD patients, and 84.0% would attend them. Considering the important role of proper diet in IBD [7,10,14,54,55] and positive patient attitudes toward further education, it would be advisable to put additional emphasis on educational programs and lectures for this population, as it could be highly beneficial for symptom management. Furthermore, for additional quality diet education, IBD patients should be encouraged to visit a licensed nutritionist and, in that manner receive, professional advice and help with a more balanced diet with less micro- and macronutrient deficiencies.

While it was shown that diet can play a pivotal role in IBD control, there are no current international recommendations on dietary patterns in IBD patients [56]. Exclusive enteral nutrition remains the number one diet for remission induction in Crohn’s disease, whereby a short-term low-FODMAP diet can benefit the patients in the acute setting and emerging evidence points to the Mediterranean diet as the best potential candidate for long term disease control [57]. Recent evidence that came in favor of the MD was from a study by Chicco et al., in which CD and UC patients were put onto a 6 month MD regimen. Their study showed a significant reduction in malnutrition and liver steatosis, as well as an improvement in disease activity and a reduction in inflammatory biomarkers [58]. Furthermore, as shown by Eder et al., the MD is not only beneficial because of its anti-inflammatory properties, but also because of its ability to prevent malnutrition and correct metabolic abnormalities which are typical in older age [24]. Furthermore, in a study by Gody et al., the authors exhibited an association between MD adherence and lower fecal calprotectin levels in UC patients after pouch surgery [59]. Moreover, Strisciuglio et al. also found significant association between adherence to MD and a low level of fecal calprotectin in children with IBD [60]. Low adherence to MD was even introduced to be a significant risk factor in development of UC in pediatric patients with IBD [61]. In light of recent findings, we believe that MD is stepping up as one of the most perspective dietary choices for IBD patients and perhaps should be brought more closely to patients via nutritional educational programs [24,35,58,59].

The limitations of our study lie in its observational design and, therefore, unfitness to determine causality. A further limitation is in the questionnaire type where we relied on the participant’s recall memory; therefore, all answers were susceptible to subjectivity and recall bias. The strength of this study lies in using a standardized method for MD adherence determination.

## 5. Conclusions

In conclusion, this is the first observational study to report very low MD adherence in IBD patients using the MDSS in the traditional Mediterranean region. The study further identifies spicy food, milk, sodas, and large meals as food types that IBD patients most associate with symptom worsening. Furthermore, results show that patients are willing to extend their nutritional knowledge if proper educational programs are organized. While there is emerging promising evidence about the benefits of MD, further prospective studies should be carried out to give clearer insight into its potential utilization in IBD management.

## Figures and Tables

**Table 1:** Baseline characteristics of study population.

Parameter	Ulcerative Colitis (*n* = 44)	Crohn’s Disease (*n* = 50)	Total (*n* = 94)	*p* *
Male gender	25 (56.8)	30 (60.0)	55 (58.5)	0.756
Age (years)	44.2 ± 14.1	37.9 ± 13.1	40.8 ± 13.9	0.027
Body weight (kg)	77.8 ± 14.3	72.7 ± 14.8	75.1 ± 14.7	0.089
Body height (cm)	176.2 ± 10.1	176.4 ± 10.0	176.3 ± 9.9	0.943
Body mass index (kg/m^2^)	25.1 ± 3.9	23.2 ± 3.72	24.1 ± 3.94	0.017
Waist circumference (cm)	90.9 ± 13.8	84.3 ± 12.1	87.4 ± 13.3	0.016
Hip circumference (cm)	100.8 ± 8.5	94.4 ± 16.3	97.4 ± 13.6	0.017
Disease duration (years)	9 (4.0–13.5)	7 (3.0–13.0)	7.7 (3.0-13.0)	0.426
IBD-associated operations (yes/no)	3 (6.8)	18 (36.0)	21 (22.3)	<0.001
Extra-intestinal manifestations	10 (22.7)	27 (54.0)	37 (39.4)	0.002
Stoma	2 (4.5)	4 (8.0)	6 (6.4)	0.681
Nutritional support (Ensure^®^, IBD modulen^®^, Vital^®^)	22 (50.0)	36 (72.0)	58 (61.7)	0.048
IV nutritional support in the last year	2 (4.5)	5 (10.0)	7 (7.4)	0.442
Decreased serum iron	26 (59.1)	28 (56.0)	54 (57.4)	0.925
Decreased vitamin D	3 (6.8)	9 (18.0)	12 (12.8)	0.129

Abbreviations: IBD, inflammatory bowel disease; IV, intravenous. Data are presented as whole numbers (%), mean ± standard deviation, or median (interquartile range; IQR); * chi-square test/Fisher’s exact test or *t*-test for independent samples/Mann–Whitney test.

**Table 2:** Laboratory and disease activity parameters.

Parameter	Ulcerative Colitis (*n* = 44)	Crohn’s Disease (*n* = 50)	Total (*n* = 94)	*p* *
Total cholesterol (mmol/L)	5.4 ± 1.43	4.3 ± 1.26	4.8 ± 1.46	<0.001
LDL cholesterol (mmol/L)	3.38 ± 1.27	2.35 ± 0.89	2.84 ± 1.21	<0.001
HDL cholesterol (mmol/L)	1.5 ± 0.48	1.28 ± 0.4	1.38 ± 0.45	0.016
Triglycerides (mmol/L)	1.12 ± 0.66	1.48 ± 1.36	1.31 ± 1.11	0.107
Total proteins (g/L)	71.8 ± 6.14	69.9 ± 7.79	70.8 ± 7.1	0.202
Albumins (g/L)	40.9 ± 4.6	38.3 ± 5.2	39.4 ± 5.1	0.008
hsCRP (mg/L)	1.8 (0.9–3.7)	2.0 (0.5–7.8)	1.85 (0.5–4.5)	0.601
Fecal calprotectin (μg/g)	138.5 (17.0–623.5)	246.5 (96.0–608.0)	208.5 (56.0–608.0)	0.128
CDAI	-	60.0 (39.0–107.0)	-	-
SES-CD	-	11.0 (5.0–20.0)	-	-
Mayo score	4.0 (2.5–7.0)	-	-	-
UCEIS	6.0 (4.0–7.0)	-	-	-
Disease activity ^†^				
remission	7 (15.9)	4 (8.0)	11 (11.7)	0.548
Mild activity	6 (13.6)	10 (20.0)	16 (17.0)	
Moderate activity	16 (36.4)	21 (42.0)	37 (39.4)	
Severe activity	15 (34.1)	15 (30.0)	30 (31.9)	
Self-assessed disease severity				
Mild disease	22 (50.0)	30 (60.0)	52 (55.3)	0.141
Moderate disease	20 (45.5)	14 (28.0)	34 (36.2)	
Severe disease	2 (4.5)	6 (12.0)	8 (8.5)	

Abbreviations: hsCRP, high-sensitivity C-reactive protein; CDAI, Crohn’s Disease Activity Index; SES-CD, Simple Endoscopic Score for Crohn Disease; UCEIS, Ulcerative Colitis Endoscopic Index of Severity; MES, Mayo Endoscopic Score; LDL, low-density lipoprotein; HDL, high-density lipoprotein. Data are presented as mean ± standard deviation or median (IQR); * *t*-test for independent samples or Mann–Whitney test; ^†^ calculated according to SES-CD and UCEIS scores.

**Table 3:** Adherence to individual and total Mediterranean Diet food groups in ulcerative colitis and Crohn’s disease groups.

Parameter	Ulcerative Colitis (*n* = 44)	Crohn’s Disease (*n* = 50)	Total (*n* = 94)	*p* *
Cereals (*n*, %)	22 (50.0)	27 (54.0)	49 (52.1)	0.856
Potato (*n*, %)	32 (72.7)	29 (58.0)	61 (64.9)	0.201
Olive oil (*n*, %)	12 (27.3)	12 (24.0)	24 (25.5)	0.899
Nuts (*n*, %)	4 (9.1)	7 (14.0)	11 (11.7)	0.533
Fresh fruit (*n*, %)	7 (15.9)	7 (14.0)	14 (14.9)	0.975
Vegetables (*n*, %)	4 (9.1)	6 (12.0)	10 (10.6)	0.745
Milk and dairy products (*n*, %)	6 (13.6)	18 (36.0)	24 (25.5)	0.025
Legumes (*n*, %)	1 (2.3)	3 (6.0)	4 (4.3)	0.619
Eggs (*n*, %)	19 (43.2)	20 (40.0)	39 (41.5)	0.918
Fish (*n*, %)	15 (34.1)	14 (28.0)	29 (30.9)	0.678
White meat (*n*, %)	17 (38.6)	16 (32.0)	33 (35.1)	0.648
Red meat (*n*, %)	20 (45.5)	28 (56.0)	48 (51.1)	0.415
Sweets (*n*, %)	23 (52.3)	38 (76.0)	61 (64.9)	0.028
Fermented beverages (*n*, %)	2 (4.5)	4 (8.0)	6 (6.4)	0.681
Total MDSS points	6.0 (5.0–7.0)	6.0 (5.0–8.0)	6.0 (5.0–7.0)	0.521 ^†^
Adherence to Mediterranean Diet	3 (6.8)	6 (12.0)	9 (9.6)	0.494

Abbreviations: MDSS, Meditteranean diet service score. Data are presented as whole numbers (%) or median (IQR); * chi-square test or Fisher’s exact test; ^†^ Mann–Whitney test.

**Table 4:** Adherence to individual and total Mediterranean Diet food groups according to disease activity by SES-CD and UCEIS scores.

Parameter	Remission/Mild Endoscopic Disease (*n* = 27)	Moderate/Severe Endoscopic Disease (*n* = 67)	Total (*n* = 94)	*p* *
Cereals (*n*, %)	15 (55.6)	34 (50.7)	49 (52.1)	0.846
Potato (*n*, %)	19 (70.4)	42 (62.7)	61 (64.9)	0.640
Olive oil (*n*, %)	7 (25.9)	17 (25.4)	24 (25.5)	0.837
Nuts (*n*, %)	3 (11.1)	8 (11.9)	11 (11.7)	0.998
Fresh fruit (*n*, %)	3 (11.1)	11 (16.4)	14 (14.9)	0.750
Vegetables (*n*, %)	2 (7.4)	8 (11.9)	10 (10.6)	0.718
Milk and dairy products (*n*, %)	6 (22.2)	18 (26.9)	24 (25.5)	0.837
Legumes (*n*, %)	1 (3.7)	3 (4.5)	4 (4.3)	0.998
Eggs (*n*, %)	13 (48.1)	26 (38.8)	39 (41.5)	0.548
Fish (*n*, %)	8 (29.6)	21 (31.3)	29 (30.9)	0.933
White meat (*n*, %)	10 (37.0)	23 (34.3)	33 (35.1)	0.992
Red meat (*n*, %)	13 (48.1)	35 (52.2)	48 (51.1)	0.896
Sweets (*n*, %)	16 (59.3)	45 (67.2)	61 (64.9)	0.626
Wine (*n*, %)	2 (7.4)	4 (6.0)	6 (6.4)	0.998
Total MDSS points	6.0 (5.0–7.0)	6.0 (5.0–7.75)	6.0 (5.0–7.0)	0.598 ^†^
Adherence to Mediterranean diet	3 (11.1)	6 (9.0)	9 (9.6)	0.713

Data are presented as whole numbers (%) or median (IQR); * chi-square test or Fisher’s exact test; ^†^ Mann–Whitney test.

**Table 5:** Digestive problems after eating food that participants connect with IBD-associated symptoms.

Parameter	Ulcerative Colitis (*n* = 44)	Crohn’s Disease (*n* = 50)	Total (*n* = 94)	*p* *
Stomach pain	27 (61.4)	34 (68.0)	61 (64.9)	0.648
Heartburn	7 (15.9)	10 (20.0)	17 (18.1)	0.806
Vomiting	2 (4.5)	1 (2.0)	3 (3.2)	0.598
Diarrhea	20 (45.5)	28 (56.0)	48 (51.1)	0.416
Blood in stool	13 (29.5)	4 (8.0)	17 (18.1)	0.008
Frequent bowel movement	12 (27.3)	5 (10.0)	17 (18.1)	0.035

Abbreviations: IBD, inflammatory bowel disease. Data are presented as whole numbers (%); * chi-square test or Fisher’s exact test.

**Table 6:** Correlation analysis between total MDSS points and different biochemical and anthropometric parameters.

Parameter	Total MDSS Points		
	Ulcerative Colitis (*n* = 44)	Crohn’s Disease (*n* = 50)	Total (*n* = 94)
	*r* * (*p*)	*r* * (*p*)	*r* * (*p*)
Total proteins (g/L)	0.286 (0.061)	−0.142 (0.325)	0.034 (0.746)
Albumins (g/L)	−0.143 (0.354)	−0.087 (0.546)	−0.125 (0.232)
Triglycerides (mmol/L)	0.069 (0.658)	−0.031 (0.831)	0.018 (0.862)
Total cholesterol (mmol/L)	0.019 (0.904)	0.101 (0.487)	0.078 (0.452)
HDL cholesterol (mmol/L)	0.389 (0.009)	0.326 (0.021)	0.312 (0.002)
LDL cholesterol (mmol/L)	0.027 (0.863)	0.057 (0.695)	0.014 (0.894)
Fecal calprotectin (µg/g)	−0.123 (0.427)	0.071 (0.622)	−0.005 (0.959)
hsCRP (mg/L)	0.042 (0.788)	0.092 (0.525)	0.066 (0.528)
Age (years)	−0.045 (0.771)	−0.091 (0.529)	−0.107 (0.304)
Body mass index (kg/m^2^)	−0.002 (0.987)	−0.175 (0.223)	−0.124 (0.235)
Waist circumference (cm)	0.032 (0.834)	−0.256 (0.072)	−0.173 (0.095)
Disease duration (years)	0.231 (0.131)	−0.049 (0.736)	0.063 (0.549)

Abbreviations: hsCRP, high-sensitivity C-reactive protein; * Spearman’s correlation coefficient.

**Table 7:** Attitudes about IBD and nutrition.

Questions	Ulcerative Colitis (*n* = 44)	Crohn’s Disease (*n* = 50)	Total (*n* = 94)	*p* *
Did you get diet information from your physician according to your illness? (yes?)	41 (93.2)	44 (88.0)	85 (90.4)	0.616
Have you visited a nutritionist to advise you on nutrition? (yes?)	5 (11.4)	11 (22.0)	16 (17.0)	0.271
Have you informed yourself on the internet about the diet related to your illness? (yes?)	7 (15.9)	7 (14.0)	14 (14.9)	0.975
Do you consider proper nutrition to have an important role in life overall? (yes?)	43 (97.7)	49 (98.0)	92 (97.9)	0.999
Do you consider proper nutrition to have an important role in your illness? (yes?)	42 (95.5)	43 (86.0)	85 (90.4)	0.166
Do you think that a better and more controlled diet could reduce your health problems? (yes?)	40 (90.9)	41 (82.0)	81 (86.2)	0.245
Do you consider educational programs on nutrition to be useful for patients? (yes?)	44 (100.0)	42 (84.0)	86 (91.5)	0.006
If educational programs on nutrition exist in your community, would you visit them? (yes?)	39 (88.6)	40 (80.0)	79 (84.0)	0.276

Data are presented as whole numbers (%); * chi-square test or Fisher’s exact test.

## References

[1] Guan Q. (2019). A comprehensive review and update on the pathogenesis of inflammatory bowel disease. J. Immunol. Res..

[2] Windsor J.W., Kaplan G.G. (2019). Evolving epidemiology of IBD. Curr. Gastroenterol. Rep..

[3] Bernstein C.N., Benchimol E.I., Bitton A., Murthy S.K., Nguyen G.C., Lee K., Cooke-Lauder J., Kaplan G.G. (2019). The impact of inflammatory bowel disease in canada 2018: Extra-intestinal diseases in IBD. J. Can. Assoc. Gastroenterol..

[4] Tadin Hadjina I., Zivkovic P.M., Matetic A., Rusic D., Vilovic M., Bajo D., Puljiz Z., Tonkic A., Bozic J. (2019). Impaired neurocognitive and psychomotor performance in patients with inflammatory bowel disease. Sci. Rep..

[5] Zivkovic P.M., Matetic A., Tadin Hadjina I., Rusic D., Vilovic M., Supe-Domic D., Borovac J.A., Mudnic I., Tonkic A., Bozic J. (2020). Serum catestatin levels and arterial stiffness parameters are increased in patients with inflammatory bowel disease. J. Clin. Med..

[6] Seyedian S.S., Nokhostin F., Malamir M.D. (2019). A review of the diagnosis, prevention, and treatment methods of inflammatory bowel disease. J. Med. Life.

[7] Rizzello F., Spisni E., Giovanardi E., Imbesi V., Salice M., Alvisi P., Valerii M.C., Gionchetti P. (2019). Implications of the westernized diet in the onset and progression of IBD. Nutrients.

[8] Renna S., Cottone M., Orlando A. (2014). Optimization of the treatment with immunosuppressants and biologics in inflammatory bowel disease. World J. Gastroenterol..

[9] Sica G.S., Biancone L. (2013). Surgery for inflammatory bowel disease in the era of laparoscopy. World J. Gastroenterol..

[10] Green N., Miller T., Suskind D., Lee D. (2019). A review of dietary therapy for IBD and a vision for the future. Nutrients.

[11] Richman E., Rhodes J.M. (2013). Review article: Evidence-based dietary advice for patients with inflammatory bowel disease. Aliment. Pharmacol. Ther..

[12] Moszak M., Szulińska M., Bogdański P. (2020). You are what you eat—The relationship between diet, microbiota, and metabolic disorders—A review. Nutrients.

[13] Mentella M.C., Scaldaferri F., Pizzoferrato M., Gasbarrini A., Miggiano G.A.D. (2020). Nutrition, IBD and gut microbiota: A review. Nutrients.

[14] Molendijk I., van der Marel S., Maljaars P.W.J. (2019). Towards a food pharmacy: Immunologic modulation through diet. Nutrients.

[15] Casanova M.J., Chaparro M., Molina B., Merino O., Batanero R., Dueñas-Sadornil C., Robledo P., Garcia-Albert A.M., Gómez-Sánchez M.B., Calvet X. (2017). Prevalence of malnutrition and nutritional characteristics of patients with inflammatory bowel disease. J. Crohns. Colitis.

[16] Cohen A.B., Lee D., Long M.D., Kappelman M.D., Martin C.F., Sandler R.S., Lewis J.D. (2013). Dietary patterns and self-reported associations of diet with symptoms of inflammatory bowel disease. Dig. Dis. Sci..

[17] Dixon L.J., Kabi A., Nickerson K.P., McDonald C. (2015). Combinatorial effects of diet and genetics on inflammatory bowel disease pathogenesis. Inflamm. Bowel Dis..

[18] Trichopoulou A., Martínez-González M.A., Tong T.Y., Forouhi N.G., Khandelwal S., Prabhakaran D., Mozaffarian D., de Lorgeril M. (2014). Definitions and potential health benefits of the Mediterranean diet: Views from experts around the world. BMC Med..

[19] Clement-Carbonell V., Ferrer-Cascales R., Zaragoza-Martí A., Ruiz-Robledillo N., Fernández-Alcántara M., Cabañero-Martínez M.J. (2018). Effects of lifestyles and the Mediterranean diet on elderly people’s quality of life according to gender. Psych. Stud..

[20] Ferrer-Cascales R., Albaladejo-Blázquez N., Ruiz-Robledillo N., Rubio-Aparicio M., Laguna-Pérez A., Zaragoza-Martí A. (2018). Low adherence to the Mediterranean diet in isolated adolescents: The mediation effects of stress. Nutrients.

[21] Khalili H., Håkansson N., Chan S.S., Chen Y., Lochhead P., Ludvigsson J.F., Chan A.T., Hart A.R., Olén O., Wolk A. (2020). Adherence to a Mediterranean diet is associated with a lower risk of later-onset Crohn’s disease: Results from two large prospective cohort studies. Gut.

[22] Papada E., Amerikanou C., Forbes A., Kaliora A.C. (2020). Adherence to Mediterranean diet in Crohn’s disease. Eur. J. Nutr..

[23] Lo C.H., Khalili H., Song M., Lochhead P., Burke K.E., Richter J.M., Giovannucci E.L., Chan A.T., Ananthakrishnan A.N. (2020). Healthy lifestyle is associated with reduced mortality in patients with inflammatory bowel diseases. Clin. Gastroenterol. Hepatol..

[24] Eder P., Niezgódka A., Krela-Kaźmierczak I., Stawczyk-Eder K., Banasik E., Dobrowolska A. (2019). Dietary support in elderly patients with inflammatory bowel disease. Nutrients.

[25] Reddavide R., Rotolo O., Caruso M.G., Stasi E., Notarnicola M., Miraglia C., Nouvenne A., Meschi T., De’ Angelis G.L., Di Mario F. (2018). The role of diet in the prevention and treatment of inflammatory bowel diseases. Acta Biomed..

[26] Aleksandrova K., Romero-Mosquera B., Hernandez V. (2017). Diet, gut microbiome and epigenetics: Emerging links with inflammatory bowel diseases and prospects for management and prevention. Nutrients.

[27] Maaser C., Sturm A., Vavricka S.R., Kucharzik T., Fiorino G., Annese V., Calabrese E., Baumgart D.C., Bettenworth D., Borralho Nunes P. (2019). ECCO-ESGAR guideline for diagnostic assessment in IBD Part 1: Initial diagnosis, monitoring of known IBD, detection of complications. J. Crohns. Colitis.

[28] Daperno M., D’Haens G., Van Assche G., Baert F., Bulois P., Maunoury V., Sostegni R., Rocca R., Pera A., Gevers A. (2004). Development and validation of a new, simplified endoscopic activity score for Crohn’s disease: The SES-CD. Gastrointest. Endosc..

[29] Sostegni R., Daperno M., Scaglione N., Lavagna A., Rocca R., Pera A. (2003). Review article: Crohn’s disease: Monitoring disease activity. Aliment. Pharmacol. Ther..

[30] Travis S.P., Schnell D., Krzeski P., Abreu M.T., Altman D.G., Colombel J.F., Feagan B.G., Hanauer S.B., Lichtenstein G.R., Marteau P.R. (2013). Reliability and initial validation of the ulcerative colitis endoscopic index of severity. Gastroenterology.

[31] Lewis J.D., Chuai S., Nessel L., Lichtenstein G.R., Aberra F.N., Ellenberg J.H. (2008). Use of the noninvasive components of the Mayo score to assess clinical response in ulcerative colitis. Inflamm. Bowel Dis..

[32] Sturm A., Maaser C., Calabrese E., Annese V., Fiorino G., Kucharzik T., Vavricka S.R., Verstockt B., van Rheenen P., Tolan D. (2019). ECCO-ESGAR guideline for diagnostic assessment in IBD Part 2: IBD scores and general principles and technical aspects. J. Crohns. Colitis.

[33] Monteagudo C., Mariscal-Arcas M., Rivas A., Lorenzo-Tovar M.L., Tur J.A., Olea-Serrano F. (2015). Proposal of a Mediterranean diet serving score. PLoS ONE.

[34] Bach-Faig A., Berry E.M., Lairon D., Reguant J., Trichopoulou A., Dernini S., Medina F.X., Battino M., Belahsen R., Miranda G. (2011). Mediterranean diet pyramid today. Science and cultural updates. Public Health Nutr..

[35] Taylor L., Almutairdi A., Shommu N., Fedorak R., Ghosh S., Reimer R.A., Panaccione R., Raman M. (2018). Cross-sectional analysis of overall dietary intake and mediterranean dietary pattern in patients with Crohn’s disease. Nutrients.

[36] Garcia-Closas R., Berenguer A., Gonzalez C. (2006). Changes in food supply in Mediterranean countries from 1961 to 2001. Public Health Nutr..

[37] Aparicio-Soto M., Sánchez-Hidalgo M., Rosillo M.Á., Castejón M.L., Alarcón-de-la-Lastra C. (2016). Extra virgin olive oil: A key functional food for prevention of immune-inflammatory diseases. Food Funct..

[38] Casas R., Sacanella E., Urpí-Sardà M., Corella D., Castañer O., Lamuela-Raventos R.M., Salas-Salvadó J., Martínez-González M.A., Ros E., Estruch R. (2016). Long-term immunomodulatory effects of a Mediterranean diet in adults at high risk of cardiovascular disease in the prevención con dieta mediterránea (PREDIMED) randomized Controlled trial. J. Nutr..

[39] Cariello M., Contursi A., Gadaleta R.M., Piccinin E., De Santis S., Piglionica M., Spaziante A.F., Sabbà C., Villani G., Moschetta A. (2020). Extra-virgin olive oil from apulian cultivars and intestinal inflammation. Nutrients.

[40] Biasi F., Deiana M., Guina T., Gamba P., Leonarduzzi G., Poli G. (2014). Wine consumption and intestinal redox homeostasis. Redox. Biol..

[41] O’Connor P.M., Lapointe T.K., Beck P.L., Buret A.G. (2010). Mechanisms by which inflammation may increase intestinal cancer risk in inflammatory bowel disease. Inflamm. Bowel Dis..

[42] Swanson G.R., Tieu V., Shaikh M., Forsyth C., Keshavarzian A. (2011). Is moderate red wine consumption safe in inactive inflammatory bowel disease?. Digestion.

[43] Siegmund S., Spanagel R., Singer M.V. (2003). Role of the brain-gut axis in alcohol-related gastrointestinal diseases—What can we learn from new animal models?. J. Physiol. Pharmacol..

[44] Peñalvo J.L., Oliva B., Sotos-Prieto M., Uzhova I., Moreno-Franco B., León-Latre M., Ordovás J.M. (2015). Greater adherence to a Mediterranean dietary pattern is associated with improved plasma lipid profile: The aragon health workers study cohort. Rev. Esp. Cardiol..

[45] Alarcón de la Lastra C., Barranco M.D., Motilva V., Herrerías J.M. (2001). Mediterranean diet and health: Biological importance of olive oil. Curr. Pharm. Des..

[46] Aggarwal D., Burns H., Mclaughlin J.T., Limdi J.K. (2015). PWE-048 dietary practices in patients with inflammatory bowel disease-food for thought. Gut.

[47] Tomar S.K., Kedia S., Upadhyay A.D., Bopanna S., Yadav D.P., Goyal S., Jain S., Makharia G., Ahuja V., Singh N. (2017). Impact of dietary beliefs and practices on patients with inflammatory bowel disease: An observational study from India. JGH Open.

[48] Patel B., Schutte R., Sporns P., Doyle J., Jewel L., Fedorak R.N. (2002). Potato glycoalkaloids adversely affect intestinal permeability and aggravate inflammatory bowel disease. Inflamm. Bowel Dis..

[49] Lobach A.R., Roberts A., Rowland I.R. (2019). Assessing the in vivo data on low/no-calorie sweeteners and the gut microbiota. Food Chem. Toxicol..

[50] Riordan A.M., Ruxton C.H., Hunter J.O. (1998). A review of associations between Crohn’s disease and consumption of sugars. Eur. J. Clin. Nutr..

[51] Hansen T.S., Jess T., Vind I., Elkjaer M., Nielsen M.F., Gamborg M., Munkholm P. (2011). Environmental factors in inflammatory bowel disease: A case-control study based on a Danish inception cohort. J. Crohns. Colitis.

[52] Octoratou M., Merikas E., Malgarinos G., Stanciu C., Triantafillidis J.K. (2012). A prospective study of pre-illness diet in newly diagnosed patients with Crohn’s disease. Rev. Med. Chir. Soc. Med. Nat. Iasi..

[53] Racine A., Carbonnel F., Chan S.S., Hart A.R., Bueno-de-Mesquita H.B., Oldenburg B., van Schaik F.D., Tjønneland A., Olsen A., Dahm C.C. (2016). Dietary patterns and risk of inflammatory bowel disease in Europe: Results from the EPIC study. Inflamm. Bowel Dis..

[54] Ruemmele F.M. (2016). Role of diet in inflammatory bowel disease. Ann. Nutr. Metab..

[55] Halmos E.P., Gibson P.R. (2015). Dietary management of IBD–Insights and advice. Nat. Rev. Gastroenterol. Hepatol..

[56] Weber A.T., Shah N.D., Sauk J., Limketkai B.N. (2019). Popular diet trends for inflammatory bowel diseases: Claims and evidence. Curr. Treat. Options Gastroenterol..

[57] Damas O.M., Garces L., Abreu M.T. (2019). Diet as adjunctive treatment for inflammatory bowel disease: Review and update of the latest literature. Curr. Treat. Options Gastroenterol..

[58] Chicco F., Magrì S., Cingolani A., Paduano D., Pesenti M., Zara F., Tumbarello F., Urru E., Melis A., Casula L. (2020). Multidimensional impact of Mediterranean diet on IBD patients. Inflamm. Bowel Dis..

[59] Godny L., Reshef L., Pfeffer-Gik T., Goren I., Yanai H., Tulchinsky H., Gophna U., Dotan I. (2019). Adherence to the Mediterranean diet is associated with decreased fecal calprotectin in patients with ulcerative colitis after pouch surgery. Eur. J. Nutr..

[60] Strisciuglio C., Cenni S., Serra M.R., Dolce P., Martinelli M., Staiano A., Miele E. (2020). Effectiveness of Mediterranean diet’s adherence in children with Inflammatory bowel diseases. Nutrients.

[61] Strisciuglio C., Giugliano F., Martinelli M., Cenni S., Greco L., Staiano A., Miele E. (2017). Impact of environmental and familial factors in a cohort of pediatric patients with inflammatory bowel disease. J. Pediatr. Gastroenterol. Nutr..

